# The Genome of Winter Moth (*Operophtera brumata*) Provides a Genomic Perspective on Sexual Dimorphism and Phenology

**DOI:** 10.1093/gbe/evv145

**Published:** 2015-07-29

**Authors:** Martijn F.L. Derks, Sandra Smit, Lucia Salis, Elio Schijlen, Alex Bossers, Christa Mateman, Agata S. Pijl, Dick de Ridder, Martien A.M. Groenen, Marcel E. Visser, Hendrik-Jan Megens

**Affiliations:** ^1^Bioinformatics Group, Wageningen University, Wageningen, The Netherlands; ^2^Department of Animal Ecology, Netherlands Institute of Ecology (NIOO-KNAW), Wageningen, The Netherlands; ^3^Research Unit Chronobiology, University of Groningen, Groningen, The Netherlands; ^4^PRI Bioscience, Plant Research International, Wageningen UR, Wageningen, The Netherlands; ^5^Department of Infection Biology, Central Veterinary Institute, Wageningen UR, Lelystad, The Netherlands; ^6^Animal Breeding and Genomics Centre, Wageningen University, Wageningen, The Netherlands

**Keywords:** winter moth, Lepidoptera, cytochrome P450, sexual dimorphism, phenology, circadian clock

## Abstract

The winter moth (*Operophtera brumata*) belongs to one of the most species-rich families in Lepidoptera, the Geometridae (approximately 23,000 species). This family is of great economic importance as most species are herbivorous and capable of defoliating trees. Genome assembly of the winter moth allows the study of genes and gene families, such as the cytochrome P450 gene family, which is known to be vital in plant secondary metabolite detoxification and host-plant selection. It also enables exploration of the genomic basis for female brachyptery (wing reduction), a feature of sexual dimorphism in winter moth, and for seasonal timing, a trait extensively studied in this species. Here we present a reference genome for the winter moth, the first geometrid and largest sequenced Lepidopteran genome to date (638 Mb) including a set of 16,912 predicted protein-coding genes. This allowed us to assess the dynamics of evolution on a genome-wide scale using the P450 gene family. We also identified an expanded gene family potentially linked to female brachyptery, and annotated the genes involved in the circadian clock mechanism as main candidates for involvement in seasonal timing. The genome will contribute to Lepidopteran genomic resources and comparative genomics. In addition, the genome enhances our ability to understand the genetic and molecular basis of insect seasonal timing and thereby provides a reference for future evolutionary and population studies on the winter moth.

## Introduction

The winter moth (*Operophtera brumata*) is an insect species that belongs to the order of Lepidoptera (butterflies, moths, and skippers). It is a member of one of the largest families, the Geometridae, containing approximately 23,000 species (Scoble 2007). The vast majority of Lepidoptera are phytophagous and many geometrid moths are considered pests. They evolved in parallel to the evolution of the flowering plants (angiosperms) during and after the Cretaceous period (Wahlberg et al. 2013). This coevolutionary process, similar to other herbivorous insect groups, involved continuous adaptation to plant allelochemicals. A popular metaphor is that of an evolutionary arms race. Among the major groups of genes involved, both in plants and insects, are the ubiquitous P450 or CYP genes (Schuler 2011). Although evolution and ecology of host-plant choice and adaptation has been studied for over a century, the underlying genetic mechanisms are poorly understood.

**Fig. 1:**
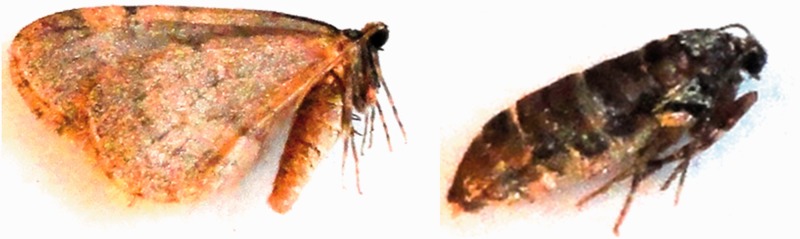
—Male (left) and female (right) winter moth. The pictures show female brachyptery in winter moth.

The winter moth is widespread in Northern Europe and Asia and after 1930 it has become an invasive pest species in North America (Elkinton et al. 2010). Previous studies have described a shift in host-range in N. America compared with native Europe (Elkinton et al. 2010). When studying host selection, the P450 gene repertoire in winter moth is of special interest, as this gene family is involved in plant secondary metabolite detoxification. Another interesting adaptation displayed by the winter moth is female brachyptery, also known as wing reduction (fig. 1) (Meyer-Rochow and Lau 2008). This feature is not uncommon in moths (described in 26 of approximately 120 families; Viloria et al. 2003) and appears to be linked to adaptation to living in woods. The high degree of convergent evolution of brachyptery in moths due to adaptive forces suggests the existence of molecular pathways that may be altered in a convergent manner. Over the years, the winter moth phenology, that is, timing of biological events, has been studied extensively. Timing of egg hatching is known to have strong fitness consequences on winter moth survival and fecundity (van Asch et al. 2007). Synchronizing with the bud burst of its host plant, the Oak (*Quercus robur*) strongly affects the survival of newly hatched larvae and has reduces fecundity at the adult stage. An evolutionary response and a restoration of synchrony between the herbivore insect and its host plant have been identified (van Asch et al. 2013), but the molecular mechanism underlying the circannual clocks in both insect and plant is still unknown. A number of genes underlying circadian (daily) rhythms and their pathways have been identified (Bradshaw and Holzapfel 2007; Pegoraro and Tauber 2011). Although evidence is accumulating, the link between circadian rhythms and circannual rhythms is yet not well understood. In *O. brumata* the influence of photoperiod is still subject of investigation (van Asch et al. 2013), and the regulation of clock genes may also be moderated by ambient temperature (Chen et al. 2007).

Although the ecology of the winter moth is well studied, the current genomic knowledge on this species is limited, because genomic resources are scarce and little is known about the genome characteristics. The chromosome number in winter moth is unknown, as was, prior to this study, its genome size. Estimated genome sizes for geometrid species range from 400 to 500 Mb, but can reach up to 1.9 Gb, for example, *Euchlaena irraria* (Gregory and Hebert 2003). Related species have about 30 chromosome pairs (*n* = 28 in *Bombyx mori*; *n* = 31 in *Biston betularia*) (Van’t Hof et al. 2013). Females are the heterogametic sex in most Lepidoptera (WZ), whereas males are the homogametic sex (ZZ) (Sahara et al. 2012). In general, the Z chromosome is larger and contains more genes (Sahara et al. 2012). At the time of analysis, five other Lepidoptera genomes had been published: *B. mori* (Bombycidae) (Mita et al. 2004), *Danaus plexippus* (Nymphalidae) (Zhan et al. 2011), *Heliconius melpomene* (Nymphalidae) (Dasmahapatra et al. 2012), *Plutella xylostella* (Plutellidae) (You et al. 2013), and *Melitaea cinxia* (Nymphalidae) (Ahola et al. 2014). Genome properties such as genome size and repeat content from these species are rather diverse, although the number of protein-coding genes seems relatively conserved. Despite the presence of multiple Lepidopteran MT genomes, there is no MT genome available in the Larentiinae subfamily to which the winter moth belongs.

Here we present an annotated genome sequence for the winter moth, which fills a major taxonomic gap in Lepidopteran comparative genomics. The genome sequence provides valuable clues and testable hypotheses for understanding the winter moth’s morphology and phenology. Moreover, the genome may provide directions to pest control. Besides describing the genomic properties in general, we discuss in detail several aspects related to winter moth biology and its ecology. First, we describe the evolution of the cytochrome P450 gene family in relation to host-plant adaptation and coevolution. Then, we analyze the genomic elements that we hypothesize to be involved in the development of female brachyptery. Finally, we explore the circadian clock genes and discuss their implications in relation to seasonal timing. In this system, the combination of experimental approaches and genome sequencing offers novel and unexplored avenues into understanding the genetics of egg-hatching timing, and in the long term, the molecular mechanisms underlying seasonal timing.

## Materials and Methods

### Sequencing Strategy

A single adult winter moth female was used for the DNA extraction. The sample was collected on an oak tree in a forest in the Netherlands in December 2012. From the extracted DNA two paired-end libraries were constructed, and sequenced with 2*250 bp reads on an Illumina MiSeq instrument, resulting in overlapping forward and reverse reads. In addition three mate pair libraries were constructed with various insert sizes (1–2, 3, and 4–5 kb) and sequenced on an Illumina HiSeq2000 instrument using 101, 7, 101 flow cycles for forward, index and reverse reads, respectively. In total, we produced 27.14 Gb of raw genomic data (table 1). A more detailed description of the sequencing procedure can be found in supplementary section S1, Supplementary Material online.

**Table 1:** Sequence Data: Raw and Preprocessed

Library	Insert Size	Raw				Preprocessed			
		# Reads (Million)	# Bases (Gb)	Read Length	Coverage	# Reads (Million)	# Bases(Gb)	Read Length	Coverage
PE400_1	−145 bp	58.8	14.5	246.5	22.7	56.1	13.8	246.4	21.6
PE400_2	−193 bp	31.2	7.8	248.2	12.1	29.7	7.4	248.2	11.5
MP1-2 kb	1–2 kb	22.9	2.3	101	3.6	14.7	1.3	86.4	2.0
MP3kb	3 kb	15.1	1.5	101	2.4	4.0	0.3	85.9	0.53
MP4-5 kb	4–5 kb	10.4	1.1	101	1.6	1.7	0.1	82.3	0.21
Total		138.5	27.1		42.4	106.1	22.9		35.8

### Assembly Strategy

The winter moth reads were filtered for adapters, primers, quality (>10), and duplicates using Fastq-mcf software (v1.1.2) (Aronesty 2011). Read pairs shorter than 19 bp were discarded. From these reads, we first assembled the MT genome using MITObim (v1.6) (Hahn et al. 2013) with the MT genome of *B. mori* as reference. Manual curation was performed where alignment errors occurred. We merged gene annotations from DOGMA (Wyman et al. 2004) and MITOS (Bernt et al. 2013) to produce a consensus annotation in line with other geometrid species. To remove reads derived from the MT genome before the assembly of the nuclear genome, we aligned all reads to the *O. brumata* MT genome and set aside pairs of which one mate or both aligned to the MT sequence. Furthermore, the reads were filtered for *Wolbachia* contamination by mapping (Bowtie2 (v2.1.0); Langmead and Salzberg 2012) to all *Wolbachia* strains available in GenBank. The mapped reads were used to assemble the *Wolbachia* genome using the Celera assembler (v8.2beta) (Miller et al. 2008). This procedure was repeated iteratively by adding the assembly to the *Wolbachia* index. The preprocessing reduced the 27.1 Gb of raw data to 22.9 Gb of clean data (table 1). Finally, these clean reads were used to build a *k*-mer graph with Jellyfish (Marcais and Kingsford 2011) for *k* = 18. The size of the winter moth genome was estimated using a *k*-mer based method (Binghang et al. 2012).

We used the stand-alone error correction method from ALLPATHS-LG (release: 50721) to correct base-calling errors in the reads (Butler et al. 2008). SeqPrep was used to merge overlapping fragment data (John 2011). A de novo assembly was performed using the Celera assembler (v8.2beta) (Miller et al. 2008). We discarded all duplicate heterozygous contigs with Haplomerger (release: 20120810) (Huang et al. 2012), described in more detail in supplementary section S2, Supplementary Material online. We used SSPACE3 for additional scaffolding with Bowtie2 (v2.1.0) (Boetzer et al. 2011; Langmead and Salzberg 2012). Gapfiller (v1.10) was used to fill gaps (parameter -d 3500 to fill overestimated gap sizes due to improperly oriented paired ends) (Boetzer and Pirovano 2012). Potential contaminants were filtered using BLASTn (v2.2.8) (dc-megablast) on the nt database; sequences with exclusively prokaryotic alignments were discarded and not used in further downstream analysis. Potential nuclear MT DNA (numts) were detected using BLASTn (v2.2.8) with the MT DNA as subject. The completeness and structural consistency were assessed using the CEGMA (v2.4) pipeline and the alignment of 457 core *Drosophila* genes (Parra et al. 2007). In addition, the proteomes of two well-annotated insect species *Drosophila melanogaster* (Adams et al. 2000) and *Tribolium castaneum* (Richards et al. 2008) were aligned to the genome using genblastA (v1.0.4) (She et al. 2009). The coverage of the alignments on the scaffolds was calculated using a custom script.

### Annotation Strategy

We estimated the repeat content of the winter moth genome by grouping the preprocessed PE400 genomic reads in repeat clusters using RepeatExplorer (Novak et al. 2013) and by estimating the single-copy region of the genome from the *k*-mer distribution (supplementary fig. S1, Supplementary Material online). A de novo repeat library was generated with RepeatModeler (v1.0.4) (Smit and Hubley 2014) using RECON (v1.0.7) (Bao and Eddy 2002), RepeatScout (v1.0.5) (Price et al. 2005), and TRF (v4.0.4) (Benson 1999). This library was used by RepeatMasker (v4.0.5) (Chen 2004) together with RepBase (Jurka 2000) for repeat annotation.

The structural annotation of protein-coding genes was done using the MAKER (v2.31.6) (Holt and Yandell 2011) pipeline with ab initio predictors SNAP (release: 2006-07-28) (Korf 2004) and AUGUSTUS (v3.0.2) (Stanke and Morgenstern 2005). We used *H. melpomene* (AUGUSTUS) and *B. mori* (SNAP) as model species in the ab initio predictors. We provided proteome data from *H. melpomene, B. mori, D. plexippus, Plutella xylostella* and *Dr. melanogaster,* and EST data from *B. mori* and *D. plexippus* as homology evidence in MAKER. We used tRNAscan-SE (v1.3.1) to identify tRNA-coding sequences (Lowe and Eddy 1997). Gene functions were assigned using BLASTp (*E* value = 0.1) (Altschul et al. 1990) on the SwissProt and TrEMBL databases (Apweiler et al. 2004). We assigned protein domains and gene-ontology terms using InterProScan (Quevillon et al. 2005) against the Superfamily (Wilson et al. 2009) and Pfam (Finn et al. 2010) protein databases. OrthoMCL (v2.0.9) was used to assign proteins to orthologous groups in the orthoMCL database (Li et al. 2003). We used SignalP (Petersen et al. 2011) to detect and WoLF PSORT (Horton et al. 2007) to localize signal peptides in the winter moth proteome. GOstat was used to find overrepresented GO terms in the secretome.

### Genomic Properties

We used two methods to estimate genomic heterozygosity. First, we identified the volume of heterozygous *k*-mers from the *k*-mer distribution and divided those by the total volume of nonheterozygous *k*-mers (supplementary fig. S1, Supplementary Material online) (Binghang et al. 2012). Second, we aligned all reads to the genome using Bowtie2 (v2.1.0) (Langmead and Salzberg 2012), variants were called using freebayes (v0.9.18-25-g5781407) (Garrison and Marth 2012) and annotated with SnpEff (v4.0) (Cingolani et al. 2012). The same alignments were used to identify potential sex-chromosomal scaffolds (supplementary file S2, Supplementary Material online). A custom script was used to identify sex-scaffolds with a minimum length of 10 kb and a single nucleotide polymorphism (SNP) density less than 1 per kilobase, and genome coverage was estimated using bedtools genomecov (Quinlan and Hall 2010). We used tBLASTn to align the *B. mori*-annotated Z-chromosomal proteins to the genome.

### Gene Family Analysis

To identify gene family contractions and expansions, we clustered the proteomes of 13 different species (12 insects, 1 mammal) into orthologous groups (supplementary table S13, Supplementary Material online) using orthoMCL. Single-copy orthologs and lineage-specific gene families were extracted using custom scripts. We identified specific insect order orthologs (supplementary fig. S4, Supplementary Material online). We built a general phylogeny from 526 single-copy orthologs (supplementary fig. S5, Supplementary Material online). Alignments were performed using ClustalW and concatenated using the Hal pipeline (Robbertse et al. 2011). A phylogenetic tree was constructed using PhyML with the LG substitution model (Guindon et al. 2009). *Operophtera brumata-*specific multicopy orthologous groups and *O. brumata* singletons were extracted using a custom script. We used GOstat (Beissbarth and Speed 2004) to identify overrepresented gene ontologies using a precomputed *O. brumata* gene ontology database. We identified gene family expansions and contractions using BadiRate (Librado et al. 2012) using the general species tree that was constructed, and with -anc, -start_value 1, and -outlier parameters. Gene family outliers were extracted using custom scripts.

To identify the P450 proteins in all species (IPR001128), we used InterProScan (Quevillon et al. 2005). Next, we used MAFFT (Katoh and Standley 2013) to align the Lepidoptera P450 proteins and FastTree2 (Price et al. 2010) to build the tree. We performed a phylogenetic analysis of the four orthologous groups containing genes homologous to the *Drosophila rdx* gene. We used MAFFT (Katoh and Standley 2013) to construct the alignment and Phyml (Guindon et al. 2010) to generate the phylogenetic tree using the LG substitution model. We used iTOL (Letunic and Bork 2007) for the visualization of the phylogenies. We used the orthoMCL groups to identify the winter moth clock genes. In addition, KEGG-KAAS (Moriya et al. 2007) was used to identify winter moth clock genes and assess the completeness of the pathway.

## Results

### The Winter Moth Genome

We have reconstructed the genome sequence of a female winter moth, yielding a total assembly size of 638 Mb, 98.9% of the estimated genome size of 645 Mb (supplementary table S1 and fig. S1, Supplementary Material online). The assembly comprises 25,801 scaffolds with an N50 scaffold length of 65.6 kb. The genome is predicted to encode 16,912 protein-coding genes. It has a GC content of 38.6% and an estimated repeat content of 53.5%. The heterozygosity rate (single individual) was estimated to be 0.72% based on the *k*-mer distribution (supplementary fig. S1, Supplementary Material online). In the assembly we find a slightly lower rate of 0.64%, corresponding to approximately 4.1 M heterozygous variants (SNP/indel) (supplementary tables S2–S4, Supplementary Material online). Based on read coverage and SNP density, we identified 875 potential sex-chromosomal scaffolds with a total length of 19.5 Mb, comparable to the *B. mori* Z chromosome (20.35 Mb) (Arunkumar et al. 2009) (supplementary file S2, Supplementary Material online). Scaffolds corresponding to the W chromosome are expected to be absent from this list, because of the high repeat content and low gene density, similar to the *B. mori* W chromosome (Fujii et al. 2010).

The genome assembly was built from 27.1 Gb of raw data (22.9 Gb after preprocessing), sequenced from five DNA libraries (table 1 and supplementary fig. S2, Supplementary Material online). Even though the fragmentation of the assembly is substantial, rigorous validation indicates that the gene space is largely covered, based on the CEGMA score and presence of related proteomes (table 2). In addition, the structural consistency with the genomic reads, that is, percentage mapped and properly paired, is high (supplementary table S5, Supplementary Material online). The high quality of the genome assembly allows for a comparison with published genomes of other Lepidoptera species.

**Table 2:** Lepidopteran Genome Properties and Validation

Species	*Operophtera brumata* (v1)	*Bombyx mori* (v2) (Xia et al. 2008)	*Danaus plexippus* (v3) (Zhan and Reppert 2013)	*Heliconius melpomene* (v1.1) (Dasmahapatra et al. 2012)	*Plutella xylostella* (v1) (You et al. 2013)	*Melitaea cinxia* (v1) (Ahola et al. 2014)
Genome assembly						
Assembly size (Mb)	638.2	431.7	248.6	269.7	394.0	393.3
Number of scaffolds	25,801	43,622	5,397	3,807	1,819	6,299
N50 scaffold size (kb)	65.6	3,717.0	715.6	277.0	737.2	258.3
Min scaffold length (bp)	502	11	300	124	1,842	1,512
Max scaffold length (Mb)	0.50	16.12	6.24	1.45	3.49	0.68
Gene annotation						
Protein-coding	16,912	14,623	15,130	12,669	18,071	16,667
InterPro domain	11,793	9,892	10,034	9,803	12,877	8,529
Genomic features						
Repeat (%)	53.5	43.6	10.2	24.9	34.0	27.9
GC (%)	38.6	38.8	31.6	32.8	38.0	32.6
Coding (%)	2.9	4.1	8.4	6.5	6.4	4.0
Intron (%)	17.7	16.3	28.1	25.8	30.8	30.5
Gene length (bp)	7,752	6,029	6,001	6,779	8,083	8,129
tRNAs	579	441	379	2,373	521	2,478
Quality control						
CEGMA partial (248)	234	240	238	234	227	208
Core *Drosophila*^a^ (%)	46.6	47.7	54.0	51.2	48.8	41.6
*Drosophila*^a^ (%)	13.0	13.4	14.4	13.4	13.0	11.2
*Tribolium*^a^ (%)	17.9	17.8	18.3	17.8	17.7	15.4

^a^Percentage of genes of which more than 90% of the sequence is found on a single scaffold.

The winter moth genome is the largest lepidopteran genome published, 48% larger than *B. mori*, its closest relative for which an assembly is available. The large genome size is not due to an increased number of protein-coding genes, which is comparable to that in other Lepidoptera, but is to a large extent explained by a higher repeat content (53.5% compared with 43.6% for *B. mori*). Long interspersed elements (mainly: CR1, RTE, L2, CRE elements) and Helitron transposons are more abundant in the *O. brumata* genome compared with *B. mori* (Osanai-Futahashi et al. 2008). Nevertheless, 48.6% of the annotated repeats remain unclassified in *O. brumata* (supplementary table S6, Supplementary Material online). For 60% of the repeat sequences in this unclassified category, we found homology in other Lepidoptera genomes.

In addition to the nuclear genome of winter moth, we have reconstructed and annotated the complete MT genome. It has a total length of 15,748 bp, and contains 13 protein-coding genes, 22 tRNA, and 2 ribosomal RNA genes (supplementary table S7 and fig. S3, Supplementary Material online). BLAST (Basic Local Alignment Search Tool) results show highest sequence identity (85%) and coverage (97%) with *Apocheima cinerarium* (Liu et al. 2014) and *Phthonandria atrilineata* (Yang et al. 2009), both geometrid moths (subfamily: Ennominae). The annotated genes are in the same order and orientation as in the mitogenomes of these geometrid moths. The A+T content is 79.97%, slightly lower than for *A. cinerarium* (80.83%) and *P. atrilineata* (81.02%). The control region (802 bp) is longer than in the other two geometrid species (*A. cinerarium*, 625 bp; *P. atrilineata*, 456 bp). In addition, we identified 185 scaffolds in the nuclear genome containing potential MT insertions (numts) (supplementary file S3, Supplementary Material online).

An interesting finding was the discovery of a *Wolbachia* infection in the sequenced individual. This bacterium commonly infects insects (Werren et al. 2008), but has not been described as an endosymbiont of winter moths. We found that 0.4% of the produced genomic reads were derived from *Wolbachia*. We assembled them into 120 scaffolds spanning 1.12 Mb, with an N50 of 15.6 kb (supplementary table S8, Supplementary Material online). The assembled sequence shows highest similarity with the wPip *Wolbachia* strain from *Culex pipiens* (Klasson et al. 2008), causing cytoplasmic incompatibility in this species. Even though further analysis was outside the scope of this study, this genome sequence could add to our understanding of the functioning and evolution of bacterial endosymbionts in insects.

### Cytochrome P450

**Fig. 2:**
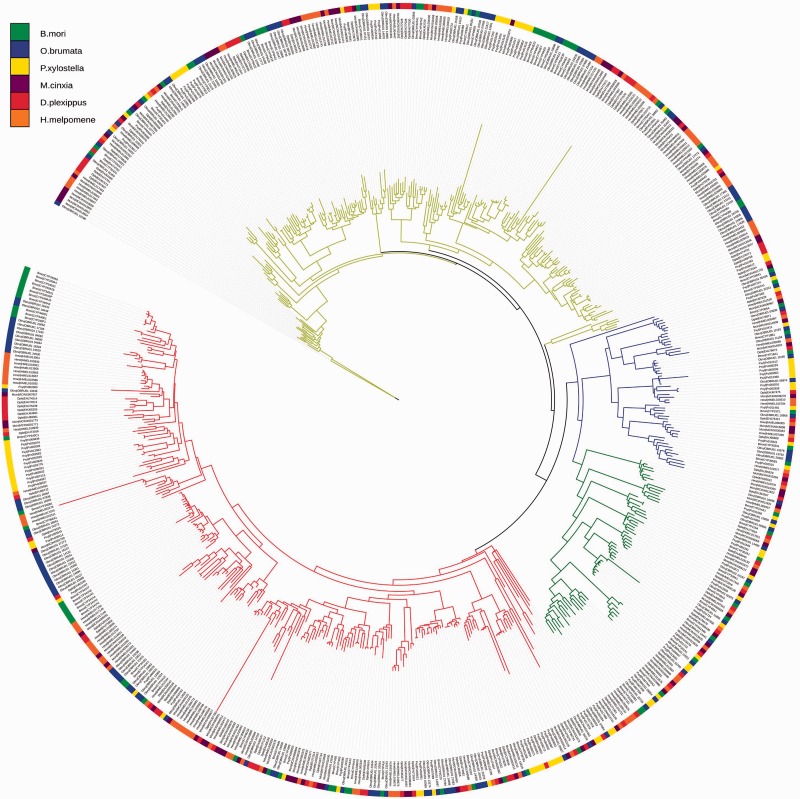
—Lepidoptera P450 gene family tree. The tree shows species-specific P450 gene family expansions in six Lepidoptera, with large winter moth expansions in CYP3 and CYP4. Branch colors represent the Cytochrome P450 clades: CYP2 (blue), CYP3 (yellow), CYP4 (red), CYP-Mito (green).

One of the main reasons to sequence the winter moth genome is to study the P450 gene family in relation to host-plant adaptation. P450 enzymes, of which large families are present in insect genomes, are involved in detoxification of plant toxins and play a central role in insecticide resistance (Feyereisen 1999). Insect P450s are subdivided into four clades, CYP2, CYP3, CYP4, and MT (Feyereisen 2006). Gene family analysis shows that winter moth contains 133 P450 genes (CYP2: 10, CYP3: 51, CYP4: 60, CYP-Mito: 12), and that members of the P450 protein family are overrepresented in winter moth-specific orthology groups. Specifically, we identified 52 *O. brumata-*specific cytochrome P450 proteins that were either unassigned or assigned to an *O. brumata-*specific group, meaning that we could not find an ortholog in any of the other five Lepidoptera (supplementary table S9, Supplementary Material online). These expansions mainly occurred in the larger CYP3 and CYP4 clades (fig. 2), with large expansions near the *B. mori Cyp340A* and *Cyp341A* genes (clade: CYP4). These expansions are likely representative for a specific detoxification gene repertoire in *O. brumata*. However, the fact that all species feed on different hosts is reflected by specific P450 gene family expansions in all species (fig. 2).

### Female Brachyptery

**Fig. 3:**
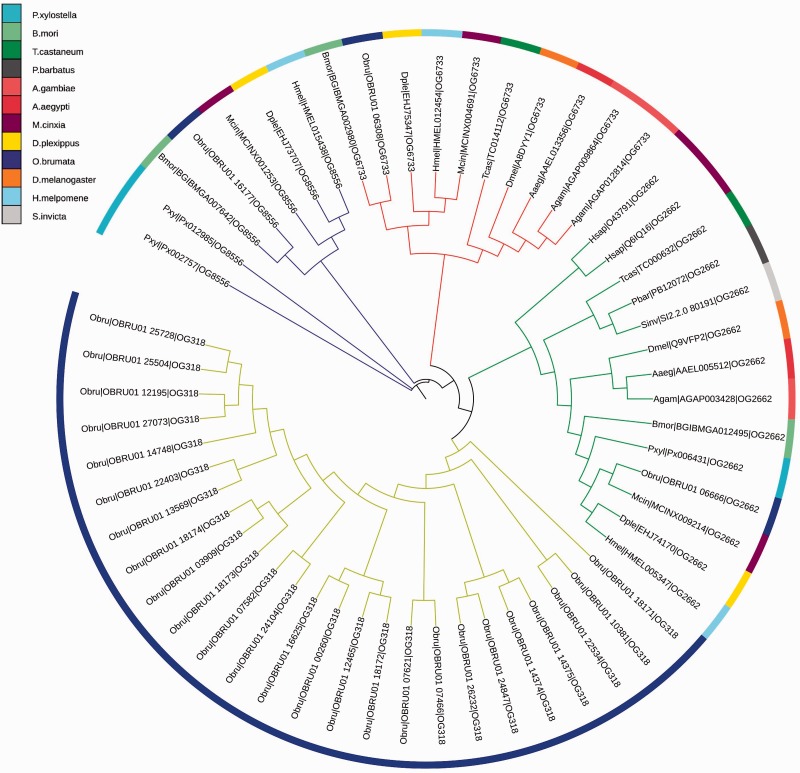
—Phylogenetic tree of the four rdx(-like) orthology groups. Orthology group OG318 (yellow) contains 25 copies of an *O. brumata* specific *rdx-like* gene. The *Drosophila* ortholog is in group OG2662. All genes have a BZB-POZ domain to interact with CUL3 except for OG6733. Only proteins in group OG2662 also contain the MATH domain.

A second striking winter moth-specific gene-family expansion is in the *rdx-like* gene family. The members of this family are organized into four orthology groups, of which one (OG318) is specific to winter moth and contains 25 proteins (fig. 3). The proteins in this group show similarity to the *Drosophila* roadkill (*rdx)* gene (Flybase: FBgn0264493). *Rdx* forms a complex with Cullin 3 (through BTB domain) and attenuates Hedgehog responses through ubiquitination of cubitus interruptus (*Ci*) (Kent et al. 2006). Hedgehog (*Hh*) signaling regulates growth and patterning in many *Drosophila* organs assumed to be similar in Lepidoptera. In wing development, *rdx* and the *CUL3*-pathway modulate the amount of *Ci* (Kent et al. 2006). However, the *Drosophila* homolog contains both a BTB/POZ (interaction with *Cul3*) domain and MATH domain (OG2662) whereas the *O. brumata* proteins in this group lack the MATH domain (supplementary table S10, Supplementary Material online). The presence or absence of this MATH domain varies among different species in the gene family (TreeFam: TF313419, PhylomeDB: phy000Z4EB). In *Drosophila*, *rdx* mutants (overexpressed) led to smaller wing sizes and differential eye morphogenesis (Kent et al. 2006; Zhang et al. 2006). This suggests that this expanded gene family may play a role in sexual dimorphism in *O. brumata*, having very short winged females and a different eye form/shape compared with males (less facets, smaller diameter, and smaller clear zone) (Meyer-Rochow and Lau 2008).

To further support this potential role in wing formation/reduction, we found four other insect species with a large *rdx-like* gene family: *Nasonia vitripennis* (Werren et al. 2010), *Lygus hesperus* (Hull et al. 2013), *Microplitis demolitor* (Burke and Strand 2012)*, Acyrthosiphon pisum* (Richards et al. 2010), supported by BLAST results (supplementary table S11, Supplementary Material online). Interestingly, male brachyptery occurs in *N. vitripennis*, and both winged and wingless morphs occur in *Ac. pisum*, triggered by overcrowding or poor food quality.

### Seasonal Timing

**Fig. 4:**
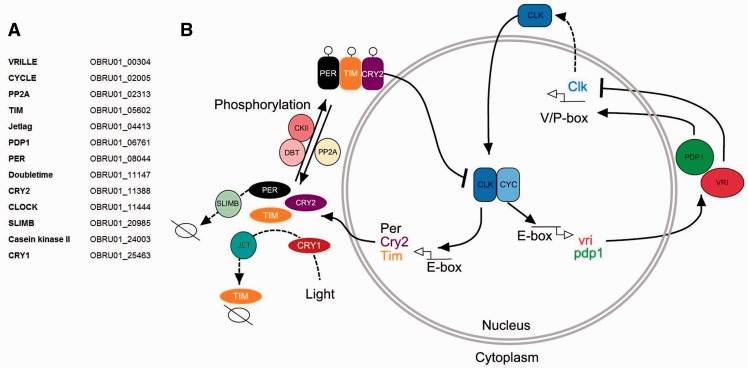
—Circadian clock mechanism in Lepidoptera. (*A*) List of clock genes, identified in the winter moth genome. (*B*) The mechanism comprises the core transcriptional/translational feedback loop (left) and the modulatory feedback loop (right). Clock (*Clk*) and Cycle (*Cyc*) form a heterodimer and drive the transcription of period (*Per*), timeless (*Tim*), and cryptrochrome 2 (*Cry2*) by binding their upstream E-box elements. *Cry2* is responsible for the inhibition of *Clk*:*Cyc* transcription, that is, its own activators. *Per* and *Tim* bind through a PAS domain. Casein kinase II (*CkII*), discs overgrown (*Dbt*), and the protein phosphatase 2A (*Pp2A*) are involved in the posttranslational modifications and activation of *Per* and *Tim*. Jetleg (*Jet*) is responsible for *Tim* degradation, modulated by *Cry1*, activated by light. Slimb signals *Per* degradation. In insects, *Per* and *Tim* are transcribed mostly during the night. *Vri* (negative) and *Pdp1* (positive) regulate the expression of *Clk* in the secondary feedback loop.

Because of its potential importance in seasonal timing, we studied the circadian clock mechanism in the winter moth genome. Our knowledge on this mechanism is based on *Drosophila*, where it comprises two transcriptional loops: A core negative transcriptional feedback loop, driving self-regulating daily rhythms and a second interlocking feedback loop (Allada and Chung 2010). *Operophtera brumata*, and all other sequenced Lepidoptera, contained all components from both loops (fig. 4 and supplementary table S12, Supplementary Material online). The critical clock genes from the core feedback loop are similar to those found in *Drosophila*; Cycle (*Cyc*), Clock (*Clk*), Period (*Per*), Timeless (*Tim*), and type-1 cryptochrome (*Cry1*). Interestingly, *O. brumata* also contains a cryptochrome 2 (*Cry2*) gene previously shown to be the main transcriptional repressor of *Cyc* and *Clk* in many other insects (Sandrelli et al. 2008), likely to be similar in winter moth. We found no evidence for clustering of this pathway in the genome. However, three of the core genes (*Clk, Cyc*, and *Per*) are located on the Z-chromosome in *B. mori*. Based on SNP density and coverage statistics we observe that *Clk* and *Per* are also located on the Z-chromosome in *O. brumata*, but Cycle is not*.* In addition, genes involved in posttranslational modifications of the *Per* and *Tim* gene are identified, as are the components from the secondary feedback loop that regulate the expression of *Clk* through Vrille (*Vri*) and *Pdp1*. These genes contain E-box elements, through which a *Clk:Cyc* complex could drive their transcription.

The *Per* and *Tim* genes are the main candidates for involvement in seasonal timing. The *Per* gene has already been found to have an effect on seasonal timing, egg-hatching, and egg-to-adult developmental time (Sauman et al. 1996; Itoh and Sumi 2000; Sandrelli et al. 2007). Also, two forms of the *Tim* gene have been found having different effects on (egg) diapause (Emerson et al. 2009). However, these effects seem to be driven by different photoperiods. In winter moth, egg-hatching is clearly influenced by temperatures, but there is no evidence of an effect of photoperiod on egg-hatching (van Asch et al. 2013). There is, however, evidence that increasing temperatures affect *Tim* expression or cause it to be alternatively spliced implicating the clock mechanism could be temperature driven (Chen et al. 2007).

## Discussion

We have successfully sequenced and assembled the winter moth genome, including its MT genome. The genome contributes to Lepidopteran phylogenomics as this is the first geometrid genome to be characterized and the first Larentiinae (which may even be a distinct family; Ounap et al. 2008) MT genome. The larger genome size compared with other Lepidoptera is to a large extent explained by its higher repeat content. However, only part of the repeats can be classified and there is little overlap with *B. mori* repeats, suggesting minor commonality within the repetitive landscape of Lepidoptera.

An interesting finding during this sequencing effort was the discovery of a *Wolbachia* infection, of which we could reconstruct a partial genome sequence, in the sequenced individual. *Wolbachia* infections are known to play a large role in sexual differentiation of hosts through cytoplasmic incompatibility, feminization, and male killing (Werren et al. 2008). Cytoplasmic incompatibility is the most abundant phenotype among sequenced *Wolbachia* genomes (Werren et al. 2008). However, the phenotype of the strain in winter moth is difficult to determine because *Wolbachia* strains can switch phenotype depending on their host (Hornett et al. 2008).

We set out with a strong interest in the P450 gene family, which is known to play an important role in detoxification of plant allelochemicals and insecticides (Schuler 2011). Gene families that confer adaptations to fast-changing environmental circumstances are known to readily expand to generate diversity in the repertoire of that adaptation. In vertebrates, for instance, immune receptor and olfactory receptor genes are well-known examples. In insects, the P450s are among gene families that have the highest number of members. The importance of these genes relative to the total number of genes in genomes has led researchers to coin the term “CYPome” (Feyereisen 2011). The number of P450 genes identified in insect genomes ranges between 56 (honey bee; Weinstock et al. 2006) and 143 (red flour beetle; Richards et al. 2008). For the winter moth we identified 133 distinct genes, at the higher end of that range. Analysis of P450 evolution compared with other Lepidoptera reveals *O. brumata* specific, and perhaps geometrid specific, expansions of P450 groups. Interestingly, the comparative analysis shows specific expansions of certain P450 for all lepidopteran genomes sequenced to date, consistent with the “periodic blooming” model of P450 evolution (Feyereisen 2011). This model states that expansions of P450 genes will occur regularly, even though they may not constitute an immediate selective advantage. However, the expanded families may confer an adaptive advantage in changing environments, for example, in adaptation to changing allelochemical composition of host plants, in host-plant preference changes or in insecticide resistance. Expanding the P450 family, irrespective of the exact gene that is being duplicated, increases the range of substances which can be oxidatively altered. Our observation is highly consistent with that model. The exact function of the majority of P450 genes in the various insect species investigated to date is very poorly understood. The midgut-specific expression of the Cyp340 and Cyp341 families (Yu et al. 2015), largely expanded in winter moth, suggests a role in detoxification of plant allelochemicals and monooxygenase activities. Our comparative analysis highlights the importance of filling in the phylogenetic gaps in herbivorous insect taxa to provide a better understanding of the evolutionary dynamics and adaptive potential of insect CYPomes.

The gene family analysis revealed an unexpected expansion in the *rdx-like* gene family, which points to a potential negative feedback mechanism in insect brachyptery. *Rdx* forms a complex with *CUL3*, leading to ubiquitination of *Ci*. *Ci* is a transcription factor regulating hedgehog (*hh*) genes, involved in many key developmental processes including wing development. It is known to have an increased expression level during wing development in other insects and studies have shown that RNA interference of *Ci* resulted in wing-reduced phenotypes (Li et al. 2009). We suggest a role for this *rdx-like* gene family in sex-specific wing-size differentiation, although the mechanism is still unclear. There may be a common regulatory basis that interacts with genes that determine gender-specific traits. For example, the transcription factor doublesex is known to mediate sexual dimorphism in insects with a male and female splice variant (Kraaijeveld 2014), and could potentially target these *rdx-like* genes. Another explanation could be sex-specific methylation, a feature described earlier in insects, but not yet linked to sexual dimorphism (D’Avila et al. 2010). The genome sequence provides insights for further experimental validation, for example, measuring and comparing expression levels in pupa stages in males and females. More elaborate experiments might entail RNA silencing during wing developmental stages in female winter moths.

Finally, with the aim to study the genomic components involved in the winter moth’s phenology, we have described the clock mechanism in winter moth as the main target pathway underlying a genetic change in response to climate change. This pathway is well conserved within insects (Sandrelli et al. 2008) and we identified all components in *O. brumata.* There is no evidence for clustering of these genes in the genome. Of the four clock genes (*Per, Clk, Cyc*, and *Pdp1*) clustered on the Z-chromosome in *B. mori*, we only find two on a sex-chromosome-related scaffold in *O. brumata* (*Per* and *Clk*), suggesting the absence of coadaptation through colocalization of this pathway. The Period gene has been shown to affect egg-hatching time in other insects such as *B. mori* (Sandrelli et al. 2007; Allada and Chung 2010). The *Per*, *Tim*, and *Cry2* genes are known to affect diapause in other insects (Xu et al. 2011; Yamada and Yamamoto 2011; Meuti et al. 2015). These genes will be the main candidates in population studies between early and late egg-hatchers to get more insight in the mechanisms behind this adaptation, or in expression studies over various temporal scales.

## Conclusion

We present the first sequenced geometrid genome, which will contribute to comparative work within Lepidoptera and insects in general. The identified (species-specific) expansions in the P450 family in all sequenced Lepidoptera could provide leads to insect detoxification of, and adaptation to, host plants and should contribute to knowledge of insecticide resistance. In addition, we imply a novel mechanism for female brachyptery supported by large *rdx-like* gene families in winter moth and in other brachypterous insects. We studied and identified the circadian clock mechanism in winter moth and argue its link to seasonal timing, providing a reference for future population studies in winter moth to unravel its rapid genetic adaptation to climate change.

## Supplementary Material

Supplementary files S1–S4, sections S1 and S2, references, figures S1–S7, and tables S1–S13 are available at *Genome Biology and Evolution* online (http://www.gbe.oxfordjournals.org/).

Supplementary Data
