# Supplementary material for: The Genome of Winter Moth (Operophtera brumata) Provides a Genomic Perspective on Sexual Dimorphism and Phenology
Source: Genome Biol Evol. 2015 Jul 29;7(8):2321–32. doi: 10.1093/gbe/evv145 (PMC4558862; doi:10.1093/gbe/evv145)
Supplement: Supplementary Data [file supp_7_8_2321__index.html]

The Genome of Winter Moth (Operophtera brumata) Provides a Genomic Perspective on Sexual Dimorphism and Phenology — Supplementary Data 

# The Genome of Winter Moth (*Operophtera brumata*) Provides a Genomic Perspective on Sexual Dimorphism and Phenology

## Supplementary Data

files

- Supplementary Data - pdf file
- Supplementary Data - xls file
- Supplementary Data - xls file
- Supplementary Data - xlsx file
