# Supplementary material for: The Genome of Winter Moth (Operophtera brumata) Provides a Genomic Perspective on Sexual Dimorphism and Phenology
Source: Genome Biol Evol. 2015 Jul 29;7(8):2321–32. doi: 10.1093/gbe/evv145 (PMC4558862; doi:10.1093/gbe/evv145)
Supplement: Supplementary Data [file supp_evv145_Supplementary_file_1.pdf]

Section S1: Sequencing strategy

DNA extraction

A single adult winter moth female was used for the DNA extraction. The sample was collected on an oak tree in a forest in the Netherlands in December 2012 and stored in ethanol at -80 °C until the DNA extraction. Total DNA from the wintermoth was extracted using the DNeasy Blood & Tissue Kit (50) (Qiagen, catalog number 69504). With a modification in step 2, the sample was lysed overnight at 56 °C, followed by RNA treatment (2 min at room temperature with 4 µl of RNase A (100mg/µL)). To clean the DNA, we performed an ethanol precipitation with 8µL 5M NaCl on 400 µL DNA and 200 µL 100% ethanol (ice-cold). We inverted 3-5 times to mix, centrifuged at 10,000 x g for 5 minutes at room temperature, and decanted all liquid. Residual ethanol was removed by air drying. Precipitated DNA was resuspended in 100 µL buffer AE. The DNA concentration was measured with the nanodrop2000 (ThermoScientific).

Overlapping library preparation and sequencing

Approximately 750 ng was sheared in a 120 µL volume using a Covaris E210 device. End repair was performed in a 200 µL total volume for 30 minutes at 30°C in a water bath then subjected to purification using AmpureXP beads (Agencourt) with 15 µL elution volume. A-tailing was performed for 30 minutes at 37°C folowed by barcoded Adapter ligation for 10 minutes at 30°C, both incubations done in a thermocycler. Adapter Ligated DNA fragments were purified using AmpureXP with 20 µL elution buffer and size selected using two slots on a 2% Agarose dye free gel (blue pippin, Sage Science). One fraction collection was done using a tight selection protocol (585 bp target, incl. 120nt of adapter sequence), whereas the second fraction collection was done using a narrow selection protocol (535 to 585 bp range, incl. 120nt of adapter sequence). Size selected fragments were Ampure XP purified and eluted in 20 µL prior to PCR amplification using 10 cycles and final library Ampure XP purification, all according to manufacturer's protocol (Illumina LT DNA sample prep guide). Libraries were quantified by Qubit fluorescence and library fragment size was analyzed by Bioanalyzer High Sensitivity DNA assay.

The two different barcoded libraries were equimolar pooled for clustering and sequencing on an Illumina MiSeq instrument using two MiSeq V2 flowcells and 2*250 cycles for Paired End sequencing plus 7 cycles for the indexing reads. De-multiplexing of resulting data was carried out using Casava 1.8 software.

Mate Pair library preparation and sequencing

Mate Pair libraries were made according to Nextera Mate Pair sample preparation Guide (Illumina) with few adaptations. Approximately 3 µg DNA was used for tagmentation in a 400 µL volume at 55°C for 30 minutes. Tagmented DNA was purified using a Zymoclean purification column and eluted in 30 µL elution buffer. Strand displacement of tagmented DNA was done for 30 minutes at room temperature. DNA was then purified using AmpureXP beads (Agencourt). Resulting yield and fragment size was analyzed using Qubit fluorescence quantification (Life Technologies) and Bioanalyzer12000 DNA chip (Agilent technologies) respectively. Approximately 750 ng tagmented repaired DNA was loaded on a 0.6% Megabase agarose gel (Bio-rad) with SYBR safe (Life Technologies) staining. After electrophoresis for 3 hours at 100 Volt, a clear smear of 1 to 9 kb DNA fragments was visible with obvious higher concentration of molecules within the shorter half. Three DNA fractions were isolated from gel, i.e. 1 to 2 kb, 3 kb and 4 to 5 kb. DNA was recovered from gel slices using Large fragment DNA recovery kit (Zymo). Of recovered fractions respectively 75, 35 and 15 ng DNA molecules was circularized for 18 hours at 30°C in a water bath, directly followed by exonuclease treatment at 37°C and inactivation at 70°C both for 30 minutes using a water bath. Remaining circularized DNA molecules were sheared using a Covaris E210 focused ultrasonicator to approximately 500 bp target size. Sheared fragments containing a biotinylated circularization adapter were enriched using M280 streptavidin dynabeads (Life technologies), followed by standard end

repair, A-tailing and barcoded adapter ligation according to manufacturer's protocol (Illumina) using a 2720 thermocycler (Life technologies) for all incubation steps. Adapter ligated fragments were then amplified using 15 PCR cycles, purified twice with ampureXP beads and resuspended in 20 µL elution buffer. The final libraries were quantified by Qubit and Bioanalyzer High Sensitivity DNA assay.

Barcoded Mate Pair libraries were equimolar pooled for clustering on one lane of a Illumina Paired End flowcell using a cBot. Sequencing was done on a Illumina HiSeq2000 instrument using 101, 7, 101 flow cycles for forward, index and reverse reads. De-multiplexing of resulting data was carried out using Casava 1.8 software.

Section S2: HaploMerger

Initially, the total assembly size (672 Mb) exceeded the genome size estimate of 645 Mb. A likely explanation is that highly heterozygous homologous chromosomal regions are split up in separate contigs during the assembly. We used HaploMerger to merge these haplotypes to produce a haploid genome assembly (Huang, et al. 2012). First, whole genome alignments with LASTZ (masked sequence) were performed using a minimum identity of 75% in order to report the alignment. Based on these alignments, overlapping contigs were identified in HaploMerger. We assumed that large contigs would be consistent with overall average genome coverage (~33X), whereas smaller contigs were more likely to include duplicate haplotypes with generally half of the expected genome coverage. The total input size of the assembly was 658.3 Mb (contigs) of which 638.2 remained after the analysis. The discarded 20.1 Mb comprised of 12,243 contigs (73,861 before, 61,618 after), on average 1,642 bp per contig. The smaller contigs had generally a lower read coverage (**Figure S6**). In addition, **Figure S7** shows a large decrease in low-coverage contigs after the HaploMerger analysis, indicating that duplicate haplotype contigs were merged.

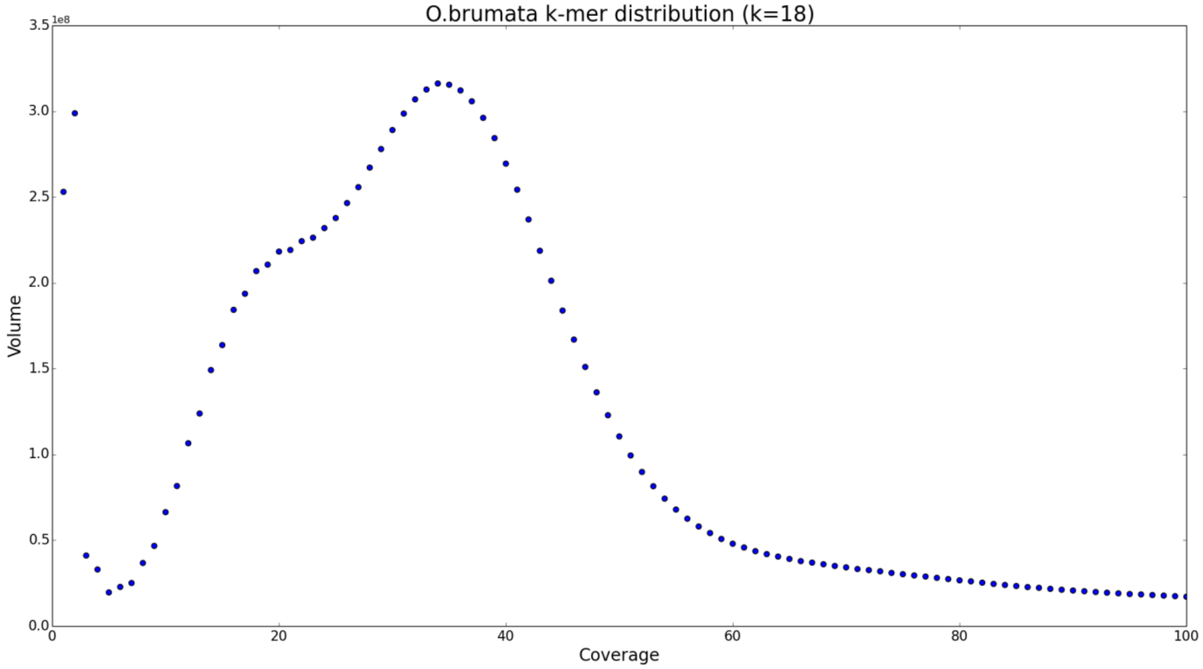

Figure S1: K-mer distribution on pre-processed data with k=18. The first peak in the distribution corresponds to *k*-mers derived from heterozygous regions, the second to *k*-mers derived from homozygous regions in the genome. The heterozygous peak ranges from ~6-24 resulting in a *k*-mer heterozygous ratio of 0.1289 (0.72 % base heterozygous ratio) meaning a SNP density of 7.19 per kb. We estimated the error *k*-mer cutoff and mean *k*-mer coverage as 5 and 34. The single copy region ranges from 6 – 65 approximately, ~47% of the total number of *k*-mers.

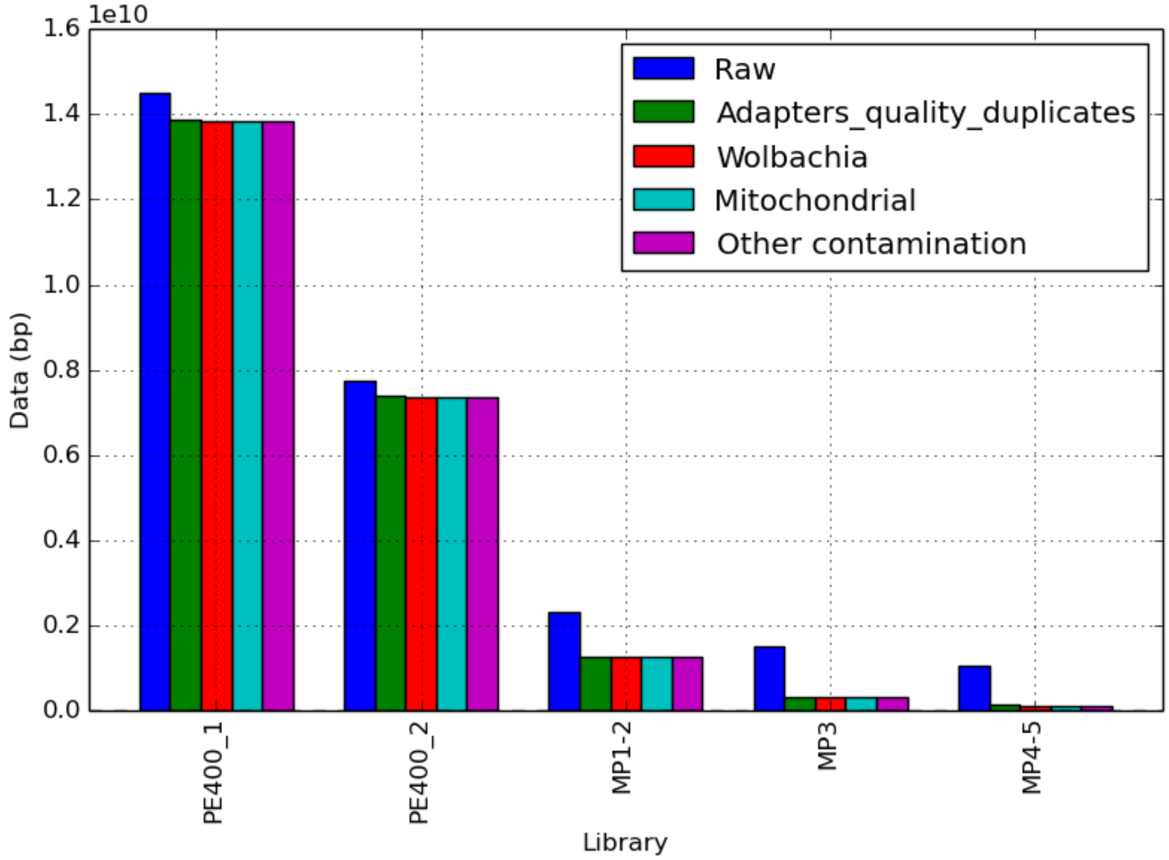

Figure S2: Data loss in each individual pre-processing step. Data was filtered for adapters, quality and duplicates. The majority of the data loss in the mate-pair data is due to the high level of clonal reads. Other contamination includes remaining prokaryotic reads.

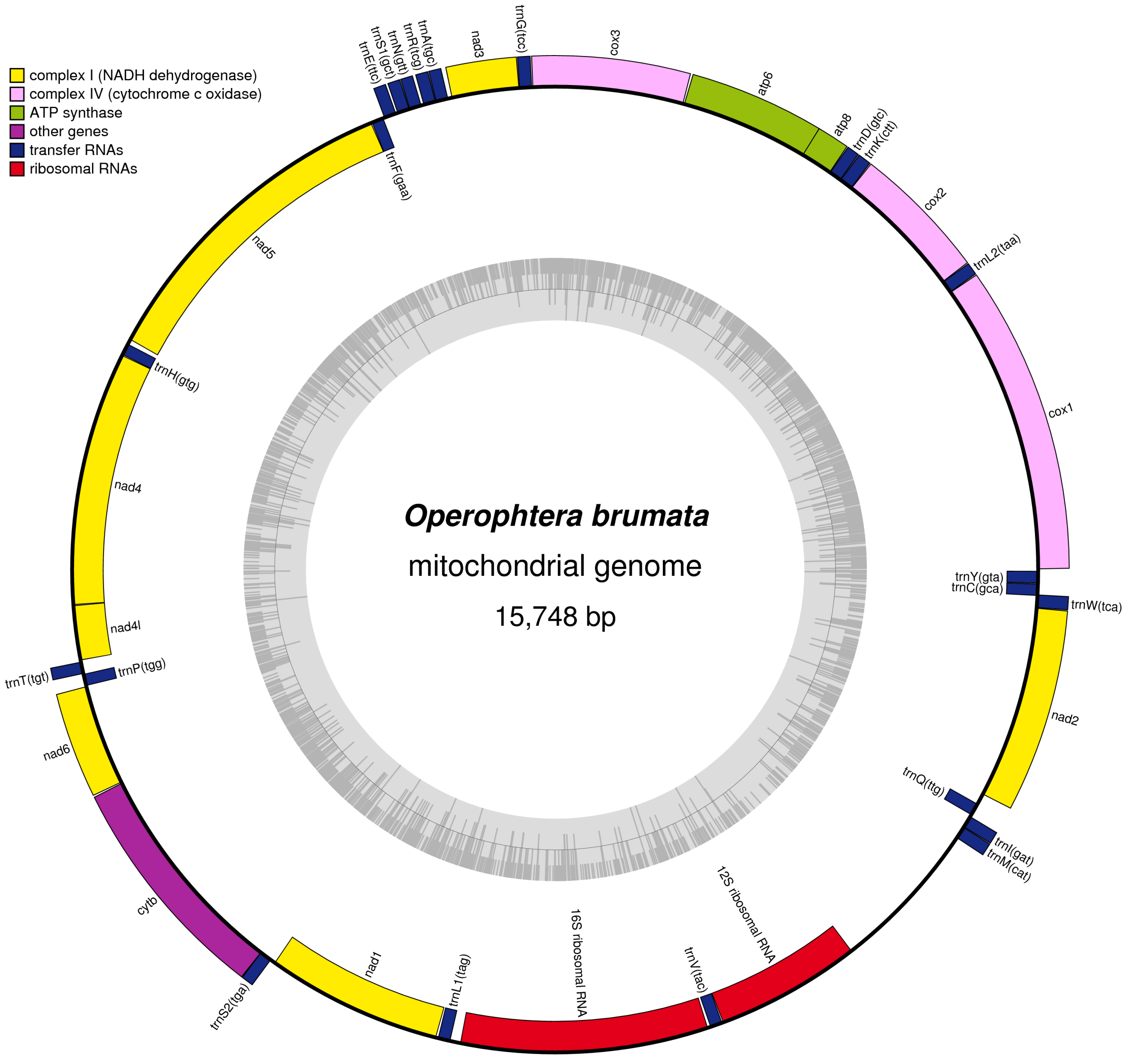

Figure S3: ***Operophtera brumata*** **mitochondrial genome.**

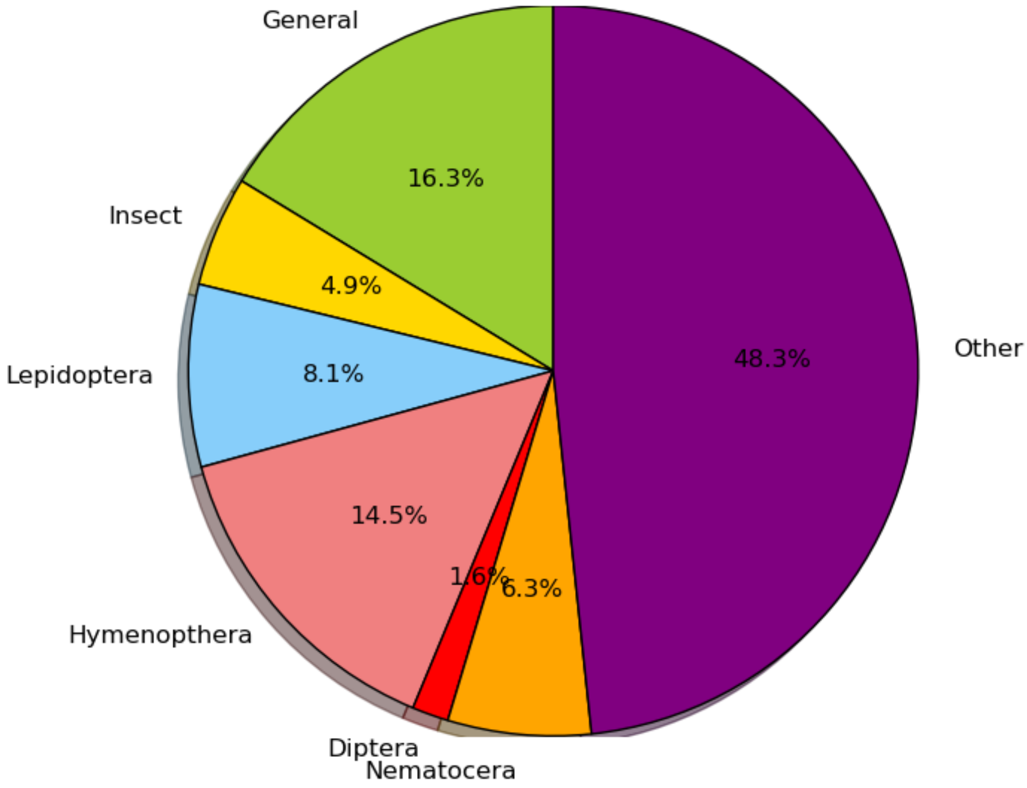

Figure S4: Orthology assessment of twelve insect proteomes and one mammalian (outgroup). The graph shows the classification of orthology groups formed from six Lepidoptera, two Hymenoptera, three Diptera, one Coleoptera, and one mammalian (*Homo sapiens*) proteome. Only multi-species groups are presented in this graph. We identified 3,052 general groups (i.e. containing proteins from all species), 915 insect-specific groups, 1,511 Lepidoptera-specific groups, 2,709 Hymenoptera-specific groups, 302 Diptera-specific groups, 1,188 Nematocera-specific groups, and 9,677 groups containing other combinations of species.

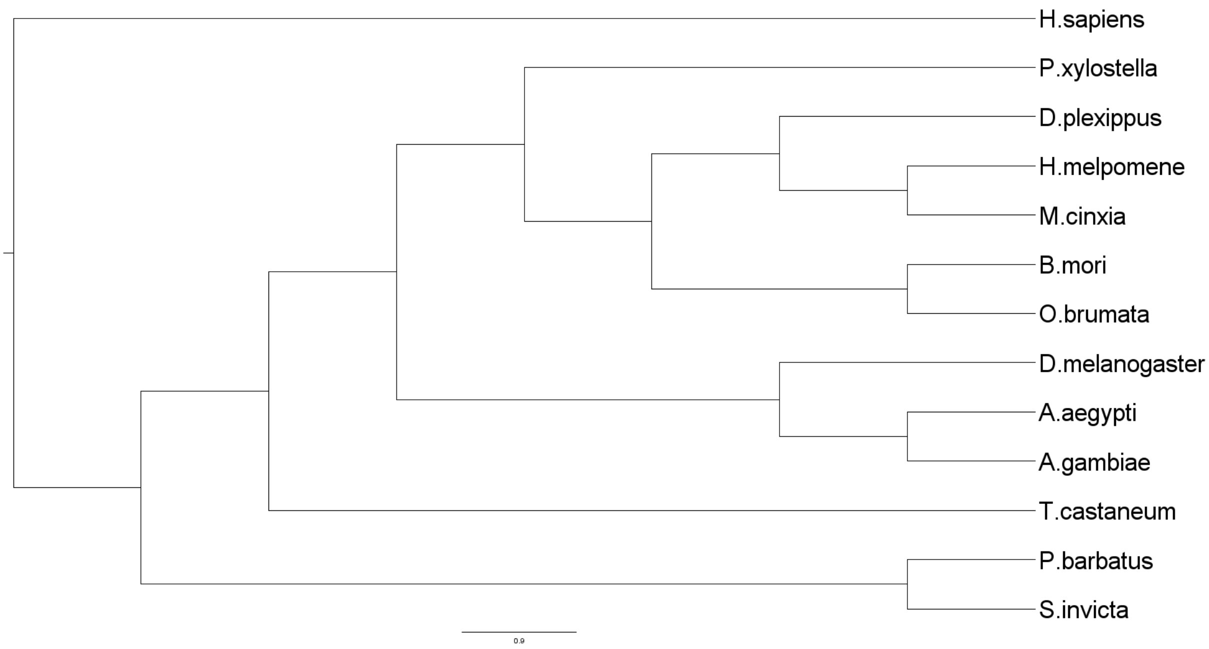

Figure S5: Phylogenetic tree based on 526 single copy orthologs. The tree shows *Bombyx mori* as closest sequenced relative.

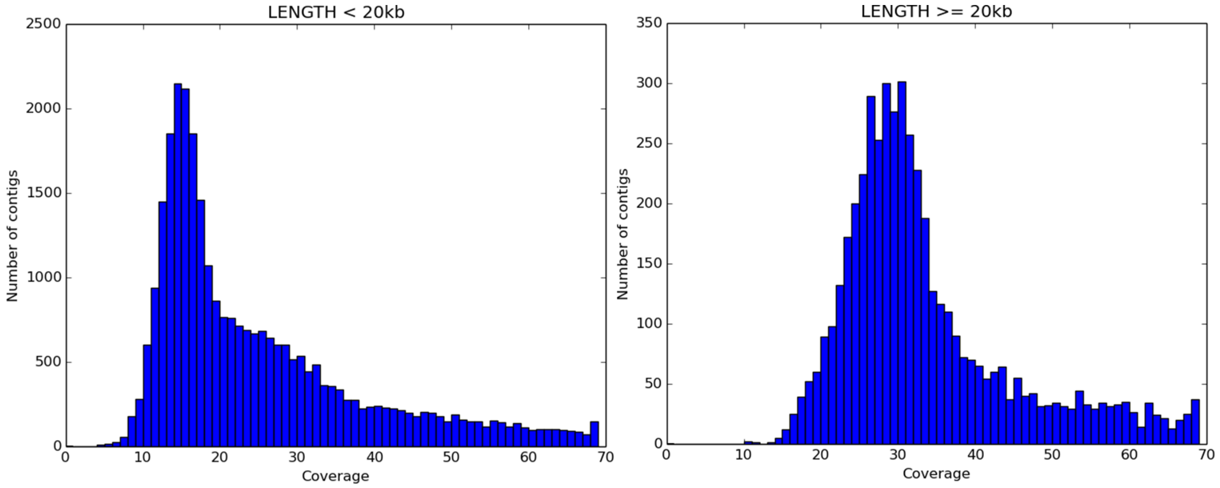

Figure S6: Histogram with the number of contigs (y) and coverage(x) for contig length < 20kb and contig length >= 20kb. Smaller size contigs <20kb have on average lower coverage compared to larger contigs (>=20kb).

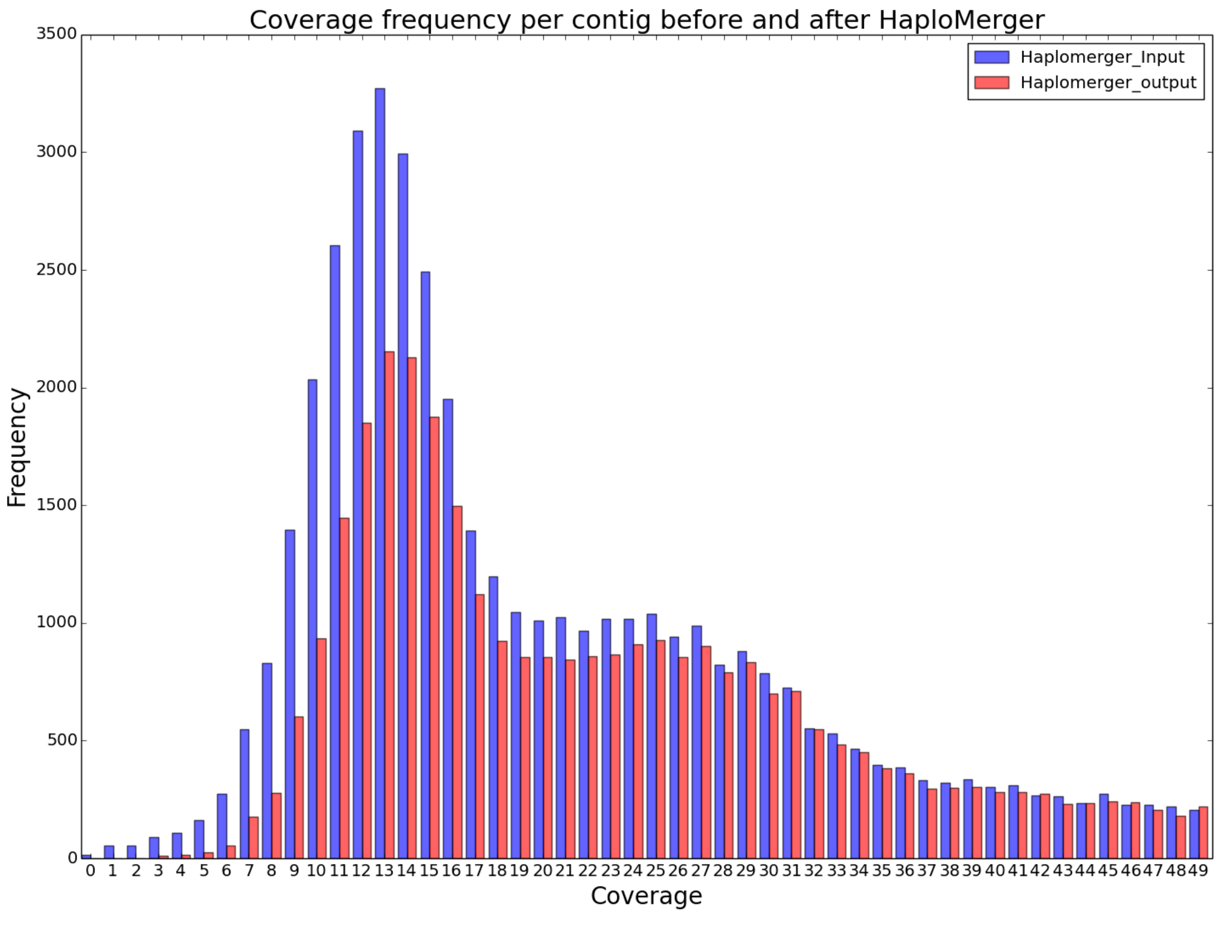

Figure S7: Coverage frequency plot before and after HaploMerger. Plot shows a large decrease in low-coverage contigs.

Table S1: Genome size estimation for K=18. In order to calculate the read coverage from the k-mer coverage we used the formula N= M /((L-K+1) / L) where M is the mean k-mer coverage, N is the actual read coverage, L is the mean read length and k is the k-mer size. (L-K+1) gives the number of k-mers created per read. Final genome size estimate is calculated by dividing the total read length by the read coverage. Another approach is to divide the total k-mers by the k-mer coverage given as the peak (M) in the k-mer distribution (Figure S1). This is equal to the BGI method using k-mers instead of actual bases.

Number of reads (million)	108.5
Mean read length	213.3
Number of *k*-mers (billion)	21.3
M	33
N	35.9
BGI (Binghang, et al. 2012) (Mb)	645.4
K-mer / M (Mb)	644.6

Table S2: Variants called in *O. brumata* genome with quality >20.

Category	Observation
Total variations	4,209,164
SNPs	3,720,548
INDELs	488,616
Homozygous SNPs	103,270
Homozygous INDELs	14,672
Heterozygous SNPs	3,617,278
Heterozygous INDELS	473,944
VAR density (1kb)	6.42
% Heterozygosity	0.64

Table S3: Distribution of variants within genomic features.

Variation	Count	Length	VAR/kb
Chromosome	3,999,186	638,087,923	6.27
Intergenic	3,096,820	507,024,664	6.11
Gene	902,366	131,063,259	6.88
Exon	87,525	18,185,505	4.81
Intron	814,841	112,877,754	7.22

Table S4: Number of variants by functional class in coding regions of the genome

Class	Count
Missense	32,917
Nonsense	849
Silent	48,574

Table S5: *O.brumata* assembly metrics.

Metric	*O. brumata* v1
Assembly size	638,087,923
Scaffold N50	2,821
Scaffold L50	65,633
Scaffold N90	10,536
Scaffold L90	13,621
Contig N50	6,070
Contig L50	28,862
Contig N90	24,599
Contig L90	5,406
GC%	38.64
Captured gaps	25,178
Maximum gap size	3,796
Mean gap size	536
Total gap length	13,489,123
% Fragment data (properly paired)	94.60 (92.70)
% Mate pair 1-2 kb (properly paired)	92.23 (71.28)
% Mate pair 3 kb (properly paired)	85.53 (59.54)
% Mate pair 4-5 kb (properly paired)	75.45 (48.44)

Table S6: ***O. brumata*** **annotated repeats.**

Class	Copies	Length (bp)	Percentage of the total repeats
**SINE**	258,674	50,559,890	14.80
**LINE**	238,419	65,339,073	19.12
Ambal	3	162	0.00
R1	2,751	952,820	0.28
CR1	80,090	20,828,845	6.10
Zorro	1	88	0.00
Rex	131	12,002	0.00
Proto1	3	153	0.00
Tad1	78	5,049	0.00
Dong	2,745	950,013	0.28
R2	68	4,883	0.00
DRE	11	631	0.00
L1	643	45,538	0.01
L2	47,792	12,371,975	3.62
LOA	2,534	847,554	0.25
Proto2	7,192	2,720,410	0.80
RTE	62,271	17,791,960	5.21
Jockey	9,864	2,874,761	0.84
I	1,251	319,596	0.09
CRE	19,079	4,785,150	1.40
Penelope	1,903	826,348	0.24
-	9	1,135	0.00
**DNA transposons**	96,334	26,966,241	7.89
Helitron	59,448	16,317,986	4.78
Harbinger	12	918	0.00
Kolobok	556	91,557	0.03
Sola	969	386,487	0.11
Crypton	1,320	721,306	0.21
Merlin	18	2,048	0.00
IS3EU	15	3,015	0.00
Zator	180	22,052	0.01
CMC	3,171	625,739	0.18
Ginger	1,156	120,051	0.04
Novosib	1,122	215,016	0.06
hAT	10,292	1,364,905	0.40
-	2,636	221,427	0.06
Dada	126	12,344	0.00
MuLE	132	53,027	0.02
Academ	1,607	825,716	0.24
Maverick	558	103,390	0.03
PiggyBac	104	13,666	0.00
TcMar	10,312	5,149,701	1.51
PIF	1,598	621,630	0.18
MULE	851	81,716	0.02
P	151	12,544	0.00
**LTRs**	19,385	4,770,062	1.40
Ginger	3	166	0.00
Gypsy	5,895	1,409,794	0.41
ERV4	30	1,664	0.00
Viper	1	131	0.00
ERV1	555	39,131	0.01
Pao	2,878	800,701	0.23
Copia	2,900	673,765	0.20
Caulimovirus	19	1,164	0.00
ERV	1	60	0.00
Copia(Xen1)	1	68	0.00
Ngaro	83	8,774	0.00
DIRS	979	221,101	0.06
ERVL	46	3,559	0.00
ERVK	5,994	1,609,984	0.47

-	513	119,546	0.03
**Satellite**	38,470	7,990,297	2.34
**Simple_repeat**	456,303	17,605,836	5.15
**Low_complexity**	44,220	2,234,354	0.65
**Unclassified**	559,269	166,035,894	48.59
**Total**	1,711,074	341,501,647	100.00

Table S7: Annotation data for the *O. brumata* mitochondrial genome (cox1 orientation). For each feature the start and stop positions and length are given in bp. Strand indicates the direction of transcription (F: forward, R:reverse).

Type	Gene	Start	Stop	Length	Strand
PCG	cytochrome c oxidase subunit 1	3	1538	1535	F
tRNA	tRNA-LEU2	1534	1600	66	F
PCG	cytochrome c oxidase subunit 2	1601	2281	680	F
tRNA	tRNA-LYS	2283	2353	70	F
tRNA	tRNA-ASP	2354	2420	66	F
PCG	ATP synthase F0 subunit 8	2421	2588	167	F
PCG	ATP synthase F0 subunit 6	2582	3259	677	F
PCG	cytochrome c oxidase subunit 3	3266	4054	788	F
tRNA	tRNA-GLY	4057	4123	66	F
PCG	NADH dehydrogenase subunit 3	4124	4477	353	F
tRNA	tRNA-ALA	4497	4562	65	F
tRNA	tRNA-ARG	4563	4628	65	F
tRNA	tRNA-ASN	4640	4704	64	F
tRNA	tRNA-SER1	4705	4770	65	F
tRNA	tRNA-GLU	4778	4843	65	F
tRNA	tRNA-PHE	4848	4913	65	R
PCG	NADH dehydrogenase subunit 5	4900	6633	1733	R
tRNA	tRNA-HIS	6655	6719	64	R
PCG	NADH dehydrogenase subunit 4	6721	8058	1337	R
PCG	NADH dehydrogenase subunit 4L	8059	8349	290	R
tRNA	tRNA-THR	8356	8419	63	F
tRNA	tRNA-PRO	8420	8484	64	R
PCG	NADH dehydrogenase subunit 6	8487	9017	530	F
PCG	cytochrome b	9022	10170	1148	F
tRNA	tRNA-SER2	10169	10234	65	F
PCG	NADH dehydrogenase subunit 1	10256	11188	932	R
tRNA	tRNA-LEU1	11196	11263	67	R
rRNA	l-rRNA	11314	12619	1305	R
tRNA	tRNA-VAL	12630	12697	67	R
rRNA	s-rRNA	12699	13469	770	R
Region	Control region	13469	14271	802	-
tRNA	tRNA-MET	14271	14338	67	F
tRNA	tRNA-ILE	14339	14403	64	F
tRNA	tRNA-GLN	14411	14479	68	R
PCG	NADH dehydrogenase subunit 2	14535	15548	1013	F
tRNA	tRNA-TRP	15549	15618	69	F
tRNA	tRNA-CYS	15611	15675	64	R
tRNA	tRNA-TYR	15676	15741	65	R

Table S8: *O. brumata Wolbachia* endosymbiont assembly statistics.

Metric	v1
Size (bp)	1,121,046
Scaffolds	120
Contigs	146
N50 index	21
N50 length	15,631
N90 index	76
N90 length	4,363
GC%	34

Table S9: *O. brumata* specific cytochrome P450 genes. No orthologs could be assigned to these P450 proteins.

Gene	Scaffold	Start	Stop	Strand	Interpro domain
OBRU01_25527	OBRU01_Sc10654	10674	12233	-1	IPR001128
OBRU01_21616	OBRU01_Sc06249	18	6945	1	IPR001128
OBRU01_12025	OBRU01_Sc01898	67644	75244	1	IPR001128
OBRU01_13174	OBRU01_Sc04285	454	1777	-1	IPR001128
OBRU01_14097	OBRU01_Sc02470	3516	7038	-1	IPR001128
OBRU01_19401	OBRU01_Sc04888	14146	33325	1	IPR001128
OBRU01_24529	OBRU01_Sc09171	6589	9814	-1	IPR001128
OBRU01_27273	OBRU01_Sc21257	73	1620	-1	IPR001128
OBRU01_18303	OBRU01_Sc04189	19598	25875	1	IPR001128
OBRU01_19888	OBRU01_Sc05077	30407	34079	-1	IPR001128
OBRU01_20151	OBRU01_Sc05287	15469	24201	-1	IPR001128
OBRU01_11193	OBRU01_Sc01692	8569	12261	1	IPR001128
OBRU01_11194	OBRU01_Sc01692	12594	14612	1	IPR001128
OBRU01_27283	OBRU01_Sc21383	733	1623	-1	IPR001128
OBRU01_27325	OBRU01_Sc23003	12	195	-1	IPR001128
OBRU01_22951	OBRU01_Sc07327	2509	15450	1	IPR001128
OBRU01_26755	OBRU01_Sc15081	1362	2094	1	IPR001128
OBRU01_14132	OBRU01_Sc05317	40657	50222	-1	IPR001128
OBRU01_22021	OBRU01_Sc06527	22885	24491	-1	IPR001128
OBRU01_16921	OBRU01_Sc07204	23630	28828	-1	IPR001128
OBRU01_25447	OBRU01_Sc11394	5044	13194	1	IPR001128
OBRU01_23243	OBRU01_Sc07602	4594	10647	1	IPR001128
OBRU01_23244	OBRU01_Sc07602	11395	16243	1	IPR001128
OBRU01_24300	OBRU01_Sc08724	13734	16202	1	IPR001128
OBRU01_07004	OBRU01_Sc01023	25808	26015	-1	IPR001128
OBRU01_20215	OBRU01_Sc05320	31416	33884	1	IPR001128
OBRU01_26433	OBRU01_Sc13356	3496	5494	1	IPR001128
OBRU01_23662	OBRU01_Sc08013	10464	17830	-1	IPR001128
OBRU01_17185	OBRU01_Sc03661	10178	33897	1	IPR001128
OBRU01_17186	OBRU01_Sc03661	37551	40807	-1	IPR001128
OBRU01_13762	OBRU01_Sc02425	1912	8211	1	IPR001128
OBRU01_19448	OBRU01_Sc04848	23957	37704	-1	IPR001128
OBRU01_25551	OBRU01_Sc10731	4	12468	-1	IPR001128;IPR013604
OBRU01_13303	OBRU01_Sc05628	23912	29384	-1	IPR001128
OBRU01_19683	OBRU01_Sc04970	21599	31533	1	IPR001128
OBRU01_21590	OBRU01_Sc05666	19588	30298	1	IPR001128
OBRU01_20852	OBRU01_Sc05475	5137	13522	-1	IPR001128
OBRU01_26067	OBRU01_Sc12028	6878	8199	1	IPR001128
OBRU01_23806	OBRU01_Sc08173	2254	10463	-1	IPR001128
OBRU01_17639	OBRU01_Sc03843	45662	48890	-1	IPR001128
OBRU01_07838	OBRU01_Sc01210	24953	26899	-1	IPR001128
OBRU01_25886	OBRU01_Sc11574	1499	5569	1	IPR001128
OBRU01_04692	OBRU01_Sc00461	12200	17438	1	IPR001128
OBRU01_24696	OBRU01_Sc11196	9299	13672	-1	IPR001128
OBRU01_07686	OBRU01_Sc00878	13048	13399	-1	IPR001128
OBRU01_27294	OBRU01_Sc21712	65	1226	1	IPR001128
OBRU01_10196	OBRU01_Sc02811	3874	5482	-1	IPR001128
OBRU01_16069	OBRU01_Sc03449	25350	55167	1	IPR001128
OBRU01_15108	OBRU01_Sc02916	23844	25143	1	IPR001128
OBRU01_15107	OBRU01_Sc02916	12991	13350	1	IPR001128
OBRU01_23424	OBRU01_Sc07769	10293	21042	1	IPR001128
OBRU01_24530	OBRU01_Sc09171	10054	16389	1	IPR001128

Table S10: Four rdx-like orthology groups including functional domain annotation. Aaeg; *Aedes aegypti*, Agam; *Anopheles gambiae*. Bmor; *Bombyx mori*, Dmel; *Drosophila melanogaster*, Dple; *Danaus plexippus*, Hmel; *Heliconius melpomene*, Hsap; *Homo sapiens*, Mcin; *Melitaea cinxia*, Obru; *Operophtera brumata*, Pbar; *Pogonomyrmex barbatus*, Pxyl; *Plutella xylostella*, Sinv; *Solenopsis invicta*, Tcas; *Tribolium castaneum*.

Species	Protein	Group	InterProScan domain	Description
Aaeg	AAEL005512-PA	OG2662	IPR011333;IPR002083; IPR008974	BTB/POZ fold;MATH;TRAF-like
Agam	AGAP003428-PA	OG2662	IPR008974;IPR011333; IPR002083	TRAF-like;BTB/POZ fold;MATH
Dple	EHJ74170	OG2662	IPR008974;IPR002083; IPR013069	TRAF-like;MATH;BTB/POZ
Pxyl	Px006431.1	OG2662	IPR013069;IPR002083; IPR011333	BTB/POZ;MATH;BTB/POZ fold
Obru	OBRU01_06666-RA	OG2662	IPR011333;IPR008974; IPR013069	BTB/POZ fold;TRAF-like;BTB/POZ
Pbar	PB12072-RA	OG2662	IPR008974;IPR013069; IPR011333	TRAF-like;BTB/POZ;BTB/POZ fold
Dmel	Q9VFP2	OG2662	IPR011333;IPR008974; IPR013069	BTB/POZ fold;TRAF-like;BTB/POZ
Mcin	MCINX009214-PA	OG2662	IPR013069;IPR011333	BTB/POZ;BTB/POZ fold
Hsap	O43791	OG2662	IPR002083;IPR008974; IPR013069	MATH;TRAF-like;BTB/POZ
Hmel	HMEL005347-PA	OG2662	IPR013069;IPR011333; IPR008974	BTB/POZ;BTB/POZ fold;TRAF-like
Tcas	TC000632-PA	OG2662	IPR002083;IPR008974; IPR013069	MATH;TRAF-like;BTB/POZ
Hsap	Q6IQ16	OG2662	IPR008974;IPR013069; IPR002083	TRAF-like;BTB/POZ;MATH
Bmor	BGIBMGA012495-TA	OG2662	IPR013069;IPR008974; IPR002083	BTB/POZ;TRAF-like;MATH
Sinv	SI2.2.0_80191	OG2662	IPR011333;IPR008974; IPR002083	BTB/POZ fold;TRAF-like;MATH
Obru	OBRU01_25728-RA	OG318	IPR013069	BTB/POZ
Obru	OBRU01_03909-RA	OG318	IPR011333	BTB/POZ fold
Obru	OBRU01_22534-RA	OG318	IPR013069	BTB/POZ
Obru	OBRU01_14374-RA	OG318	IPR013069	BTB/POZ
Obru	OBRU01_00260-RA	OG318	IPR013069	BTB/POZ
Obru	OBRU01_18171-RA	OG318	IPR011333	BTB/POZ fold
Obru	OBRU01_18173-RA	OG318	IPR011333	BTB/POZ fold
Obru	OBRU01_27073-RA	OG318	IPR011333	BTB/POZ fold
Obru	OBRU01_18172-RA	OG318	IPR011333	BTB/POZ fold
Obru	OBRU01_12465-RA	OG318	IPR011333	BTB/POZ fold
Obru	OBRU01_10381-RA	OG318	IPR013069	BTB/POZ
Obru	OBRU01_22403-RA	OG318	IPR011333	BTB/POZ fold
Obru	OBRU01_14748-RA	OG318	IPR013069	BTB/POZ
Obru	OBRU01_26232-RA	OG318	IPR013069	BTB/POZ
Obru	OBRU01_07466-RA	OG318	IPR013069	BTB/POZ
Obru	OBRU01_07582-RA	OG318	IPR011333	BTB/POZ fold
Obru	OBRU01_13569-RA	OG318	IPR013069	BTB/POZ
Obru	OBRU01_16625-RA	OG318	IPR011333	BTB/POZ fold
Obru	OBRU01_24104-RA	OG318	IPR011333	BTB/POZ fold
Obru	OBRU01_25504-RA	OG318	IPR011333	BTB/POZ fold
Obru	OBRU01_18174-RA	OG318	IPR011333	BTB/POZ fold
Obru	OBRU01_14375-RA	OG318	IPR011333	BTB/POZ fold
Obru	OBRU01_07621-RA	OG318	IPR013069	BTB/POZ
Obru	OBRU01_24847-RA	OG318	IPR011333	BTB/POZ fold
Obru	OBRU01_12195-RA	OG318	IPR013069	BTB/POZ
Obru	OBRU01_06308-RA	OG6733	IPR013069	BTB/POZ
Agam	AGAP009864-PA	OG6733	-	-
Agam	AGAP012814-PA	OG6733	-	-
Aaeg	AAEL013356-PA	OG6733	-	-
Bmor	BGIBMGA002980-TA	OG6733	-	-
Dmel	A8DYY1	OG6733	-	-
Dple	EHJ75347	OG6733	-	-

Hmel	HMEL012454-PA	OG6733	-	-
Mcin	MCINX004691-PA	OG6733	-	-
Obru	OBRU01_06308-RA	OG6733	-	-
Tcas	TC014112-PA	OG6733	-	-
Bmor	BGIBMGA007642-TA	OG8556	IPR013069	BTB/POZ
Mcin	MCINX001253-PA	OG8556	IPR011333	BTB/POZ fold
Pxyl	Px002757.1	OG8556	IPR013069	BTB/POZ
Obru	OBRU01_16177-RA	OG8556	IPR013069	BTB/POZ
Dple	EHJ73707	OG8556	IPR011333	BTB/POZ fold
Hmel	HMEL015438-PA	OG8556	IPR011333;IPR013069	BTB/POZ fold;BTB/POZ
Pxyl	Px012985.1	OG8556	IPR013069	BTB/POZ

Table S11: Blast hits (nr) for *O. brumata* rdx-like genes from all four orthologous groups. For each hit, the group with the lowest E-value is presented.

Taxonomy	Order	Species	Hits	OG2662	OG8556	OG6733	OG318
7425	Hymenoptera	*Nasonia vitripennis*	**105**	26	15	54	10
30085	Hemiptera	*Lygus hesperus*	**95**	33	11	51	0
69319	Hymenoptera	*Microplitis demolitor*	**63**	8	14	39	2
7029	Hemiptera	*Acyrthosiphon pisum*	**57**	5	4	24	24
7070	Coleoptera	*Tribolium castaneum*	31	5	16	10	0
7227	Diptera	*Drosophila melanogaster*	24	2	17	5	0
7370	Diptera	*Musca domestica*	23	3	9	11	0
7176	Diptera	*Culex quinquefasciatus*	22	2	7	12	1
13686	Hymenoptera	*Solenopsis invicta*	19	6	7	6	0
103372	Hymenoptera	*Acromyrmex echinatior*	19	5	10	4	0
136037	Isoptera	*Zootermopsis nevadensis*	18	1	7	6	4
610380	Hymenoptera	*Harpegnathos saltator*	18	7	5	6	0
77166	Coleoptera	*Dendroctonus ponderosae*	16	1	11	4	0
28588	Diptera	*Bactrocera cucurbitae*	15	1	9	3	2
27457	Diptera	*Bactrocera dorsalis*	15	1	9	3	2
7462	Hymenoptera	*Apis dorsata*	14	3	8	3	0
7222	Diptera	*Drosophila grimshawi*	13	1	10	1	1
46245	Diptera	*Drosophila pseudoobscura*	13	1	11	1	0
13037	Lepidoptera	*Danaus plexippus*	13	2	6	5	0
7091	Lepidoptera	*Bombyx mori*	13	5	4	4	0
7460	Hymenoptera	*Apis mellifera*	13	5	5	3	0
180454	Diptera	*Anopheles gambiae*	12	2	8	2	0
121224	Phthiraptera	*Pediculus humanus*	12	2	6	2	2
7260	Diptera	*Drosophila willistoni*	11	1	6	3	1
121845	Hemiptera	*Diaphorina citri*	11	2	3	6	0
132113	Hymenoptera	*Bombus impatiens*	11	2	6	3	0
7213	Diptera	*Ceratitis capitata*	11	0	7	3	1
7217	Diptera	*Drosophila ananassae*	11	1	8	2	0
7159	Diptera	*Aedes aegypti*	11	1	8	2	0
7230	Diptera	*Drosophila mojavensis*	11	1	6	3	1
30195	Hymenoptera	*Bombus terrestris*	10	1	6	3	0
7244	Diptera	*Drosophila virilis*	10	1	6	2	1
104421	Hymenoptera	*Camponotus floridanus*	9	2	4	3	0
443821	Hymenoptera	*Cerapachys biroi*	9	1	5	2	1
143995	Hymenoptera	*Megachile rotundata*	8	2	3	3	0
43151	Diptera	*Anopheles darlingi*	8	1	5	2	0
7234	Diptera	*Drosophila persimilis*	8	0	7	1	0
7220	Diptera	*Drosophila erecta*	7	0	5	1	1
74873	Diptera	*Anopheles sinensis*	6	1	4	1	0
7240	Diptera	*Drosophila simulans*	6	0	5	1	0
7238	Diptera	*Drosophila sechellia*	6	0	4	1	1
7463	Hymenoptera	*Apis florea*	6	1	4	1	0
7245	Diptera	*Drosophila yakuba*	5	0	3	1	1
30069	Diptera	*Anopheles stephensi*	2	0	2	0	0
329032	Hemiptera	*Riptortus pedestris*	1	1	0	0	0
7229	Diptera	*Drosophila miranda*	1	0	1	0	0
36987	Isoptera	*Coptotermes formosanus*	1	0	0	1	0
7165	Diptera	*Anopheles gambiae*	1	0	1	0	0

Table S12: *O. brumata* clock genes and *Bombyx mori* orthologs. The last two columns represent the SNP density and mean coverage for the corresponding *O. brumata* scaffold.

Gene	*O. brumata* protein	Interpro-Scan domain identifier	Ortho-logy group	*B. mori* ortholog	*B. mori* locus	SNP density	Mean cov-erage
VRI	OBRU01_00304	IPR004827	OG6538	BGIBMGA013421	Chr27:4490487-4500640	5.2	34.3
CYC	OBRU01_02005	IPR011598	OG1939	BGIBMGA003870	Chr1:22022407- 22117405	6.4	31.8
PP2A	OBRU01_02313	IPR004843; IPR029052	OG3715	BGIBMGA007460	Chr3:3668244- 3689014	4.8	31.2
TIM	OBRU01_05602	IPR006906	OG6006	BGIBMGA006226	Chr4:8449049- 8472466	5.4	35.2
JET	OBRU01_04413	-	OG6179	BGIBMGA007348	Chr3:1652897- 1656428	1.2	39.4
PDP1	OBRU01_06761	IPR004827	OG5114	BGIBMGA003874	Chr1: 22204769- 22297686	5.4	28.1
PER	OBRU01_08044	IPR000014; IPR013767	OG5596	BGIBMGA000486	Chr1:12956618- 13004501	1.7	20.4
DBT	OBRU01_11147	IPR011009; IPR000719	OG6809	BGIBMGA007304	Chr17: 7197366- 7208585	0.4	31.6
CRY2	OBRU01_11388	IPR005101; IPR006050	OG421	BGIBMGA007789	Chr15:9097906- 9117412	3.4	26.0
CLK	OBRU01_11444	IPR000014	OG2652	BGIBMGA000498	Chr1:6617152-6658312	0.1	21.3
SLIMB	OBRU01_20985	IPR017986; IPR001680; IPR022048	OG2886	BGIBMGA012206	Chr24: 16923175-16931013	4.3	30.8
Casein kinase II	OBRU01_24003	IPR000719; IPR011009	OG2514	BGIBMGA003474	Chr5:11364679- 11369751	0.9	42.5
CRY	OBRU01_25463	IPR006050	OG7553	BGIBMGA007140	Chr17:14889290- 14890272	3.5	19.0

Table S13: Orthology. Orthology assessment of twelve insect and one mammalian species. In total, 22,895 orthologous groups were created with 18,726 multi-species groups and 4,133 single species groups.

Organism	Order	Superfamily	#Proteins	# groups with *O. brumata*	# proteins in *O. brumata* groups
O.brumata	Lepidoptera	Geometroidea	16,912	-	-
B.mori	Lepidoptera	Bombycoidea	14,623	8,780	10,206
D.plexippus	Lepidoptera	Papilionoidea	16,254	9,021	10,925
H.melpomene	Lepidoptera	Papilionoidea	12,829	8,395	9,604
M.cinxia	Lepidoptera	Papilionoidea	16,674	8,418	9,840
P.xylostella	Lepidoptera	Yponomeutoidea	18,073	7,541	11,029
D.melanogaster	Diptera	Ephydroidea	13,907	6,148	7,444
A.aegypti	Diptera	Culicoidea	15,696	6,232	7,995
A.gambiae	Diptera	Culicoidea	12,828	6,239	7,154
P.barbatus	Hymenoptera	Vespoidea	17,189	6,436	7,477
S.invicta	Hymenoptera	Vespoidea	16,522	5,839	6,738
T. castaneum	Coleoptera	Tenebrionoidea	16,526	6,698	8,267
H.sapiens	Primates	Hominidae	20,249	5,181	8,699

References

Binghang L, et al. 2012. Estimation of genomic characteristics by analyzing k-mer frequency in de novo genome projects. arXiv:1308.2012 [q-bio.GN].
Huang SF, et al. 2012. HaploMerger: Reconstructing allelic relationships for polymorphic diploid genome assemblies. Genome Research 22: 1581-1588. doi: DOI 10.1101/gr.133652.111
